# Orthopædic Surgery—Report of a Case of Congenital Deformity Removed by Tenotomy

**Published:** 1866-08

**Authors:** J. F. Miner


ART. II.—*Orthopædic Surgery—Report of a case of congenital deformity removed by tenotomy.* By J. F. Miner, M. D.

Orthopædic surgery is so largely in the hands of a few practitioners, and so little understood and appreciated by the great majority of physicians, who might be eminently capable and successful in this department of practice, that attention cannot be too often directed to results and the manner of obtaining them. It is not infrequent to see cases wholly neglected or absurdly treated by physicians eminently capable in the general practice of medicine and surgery. Deformities may not all be removed, may not be removed perfectly in but few instances, but may almost always be greatly improved, and in every instance properly treated. A great many extravagantly represented cases have been published, and it is quite possible that some mistakes and misapprehensions have been caused by elaborate reports upon the curability and perfect restoration of spinal curvature, or the perfect results in cases of talipes and other tendinous contractions. However this may be, and it is certain we are somewhat exposed to the charge of extravagance and exaggeration, yet there is just ground for considerable pretention, and a truthful and fair representation will entitle the profession to credit and praise for the progress which has been made within the past few years in understanding and managing deformities. Cases treated with ordinary means, that is, with appliances accessible to all, especially merit attention and fair representation; costly appliances or expensive treatment in private hospitals, whatever be the results, and it is possible they are not better, interest the general practitioner and the majority of sufferers much less. Machines are often manufactured and worn for relief of spinal deviations, club-foot deformities, muscular or tendinous contractions, etc., etc., which are instruments of torture, rather than relief or cure. These instruments are mostly made to fit some theory, applied often by an inexperienced practitioner, and abandoned after imperfect trial, having done less harm from being poorly tested. Some men have no mechanical gift whatever, and should never attempt anything in this department of surgery; it is not possible for them to make the manipulations of surgery with success and satisfaction; they understand its theory and the principles of its practice, they are often safe advisers, but they cannot adapt their means to a desired object. After excluding these, as unsuited for the undertaking, it may be said of the others, that most cases of deformity can be successfully treated by them and generally by the means within their reach, provided they devote enough attention to the subject to become familiar with the principles upon which successful practice is based. Deformity from defieient muscular action or relaxation, is the most incurable form of disease, while that arising from congenital or acquired contraction, excessive or unnatural muscular action is perhaps most susceptible of cure. When muscles do not act, and the will seems to have but imperfect control, the case is unpromising, and little may be expected except from mechanical support; on the other hand, contracted tendons may be lengthened almost at pleasure, and the parts placed in normal position will at length possess almost natural form and motion.

There is a common practice with some good orthopædic surgeons, after division of contracted tendons, to delay dressing for a day or two until the irritation of operation has abated, and then bring the parts into normal position, and thus retain them. A few years since it was an almost universal practice, to make what was called temporary dressing of fractured bones, and not attempt to perfectly adjust the fragments until the first symptoms of inflammation had abated. Upon this same principle a club-foot or a contracted tendon elsewhere, is operated upon—divided, and allowed to remain three or four days, and then placed with great pain, in desired position. Upon this point there are differences of opinion, but it is believed that no harm can grow out of immediate adjustment in all operations for relief of deformities by division of tendons; that it is not only proper but very desirable to place immediately in natural position not only a fractured bone, but any deformed part it may be attempted to restore. When the surgeon operates for talipes and is three or four days before he makes retentive dressing—before he places the foot in normal position, and attempts to retain it, he must *not* be greatly disappointed if after the *second edition* of his operation, itself more painful than the first, he finds the deformity scarcely improved by his attempts. It is perhaps not quite certain that such delay will insure failure, but it is certainly quite unnecessary. It is not probable but that most fractured bones will unite if undressed and unadjusted for several days, but it is certain that if bones are fractured the processes of repair commence immediately, and placing them in contact and in natural position, removes the causes of inflammation and allows the restoration to commence more early and to progress more rapidly. The analogy between these two cases may not hold in all respects, but there are striking points of resemblance, and the one illustrates important principles of practice in the other.

In some cases of congenital deformity all the muscles and tendons of the parts favor the mal-position, are made to conform to it. One tendon may appear to be mainly in fault, but its division will reveal contractions in others, and oftentimes severel tendons will require division before the desired result is obtained. The following remarkable case of congenital deformity will illustrate this point and show important principles to be observed in operations made in similar cases:

M. Busher of Buffalo, aged 3 years, was presented for examination and operation May 26, 1866, with the following condition: Was well developed and healthy, of full natural size, well nourished in every respect, bright and active. The thighs were drawn inwards so closely that the mother had always experienced difficulty in separating them for washing, dressing, etc., etc., and were flexed strongly upon the abdomen by contraction of the sartorius, psoas, iliacus, and adductor muscles. The legs were flexed upon the thigh by contraction of the flexor muscles, and the feet extended by the shortened tendon of the gastrocnemii and solei muscles.

On account of this condition the child had never stood or walked, though the legs still retained their natural size and apparent strength.

The operation practiced for relief of this deformity consisted in dividing the tendons of all the contracted muscles while the child was under the influence of chloroform. From the great number of muscles the undertaking appeared rather formidable to be made at one time, and did prove by sympathetic disturbance that it was, for the little sufferer, a fearful expedient. All the tendons, however, appearing manifestly shortened upon both sides, were at the same time divided, and the legs brought down, first by the extension of weights over pullies; two days later, from dissatisfaction with the operation of this extension, it was exchanged for a splint, made of sole leather, cut so as to separate the legs and at the same time straighten them perfectly. This leather splint was after the style of the wire splint figured in standard works for treatment of hip-joint disease, was made to fit closely the back of the hips and legs throughout the entire length, and thus they were retained immovably for two weeks, until the wounds of operation were healed, when it was found that the legs remained in their new position remarkably well, and soon that the child could not only stand erect, but walk with the aid of a hand to steady its motions. It has since learned to walk, and though it will never have full natural scope and flexibility of motion, yet will be able to stand erect and walk with great respectability of appearance and usefulness of action.

This child was examined by several very experienced physicians who appear from the account of the parents to have entertained very different and I may say conflicting opinions as to the nature of the malady. It may be interesting as showing how the best and most careful observers sometimes disagree, to state that the family physician, experienced and capable, in general practice, regarded this want of power to use the legs as arising from disease of the spine; another physician, eminent in his department, told the parents it was paralysis, and that the trouble was probably in the head; both assured them that nothing could be done for the child. A third, with different view, prescribed quinine for a long time, and better yet, made attempts to remedy the trouble by extension. Many had remedies which they thought might "strengthen the child a little," and advised nutritious food, tonics, etc., while quite a number of irregular practitioners prescribed liniments, plasters, salves, washes, etc., etc. All this goes to show how important it is that attention be directed to these cases and the general symptoms and appearances described. It is not very remarkable that some different views should be entertained as to the nature of such a case, by men only accustomed to see and prescribe for ordinary disease, but every surgeon in any degree familiar with such conditions should not make incorrect diagnosis; the instinct almost, should at once tell him that his art is capable of affording relief. It is never safe, however, in these operations to promise too much; they are not in the after dressings perfectly under the control of the physician, and if they were, perfect recovery would hardly be attained. Almost all deformities from contracted muscles, admit of greater or less relief, and the earlier, congenital mal-positions are corrected, the more perfect the result. Sometimes the question is asked, how old should a child be before such operations are made? The question should rather be, how young? for every experienced surgeon understands with what ease the tender yielding tissues of a new born infant may be moulded and changed. I have often thought that division of tendons would rarely be required, if attempts to rectify abnormal positions were made immediately after birth.

